# Comparative Safety Signal Assessment of Hospitalization Associated With the Use of Atypical Antipsychotics

**DOI:** 10.3389/fpsyt.2022.917351

**Published:** 2022-06-06

**Authors:** Ismaeel Yunusa, Chengwen Teng, Ibraheem M. Karaye, Emily Crounse, Saud Alsahali, Nasim Maleki

**Affiliations:** ^1^Department of Clinical Pharmacy and Outcomes Sciences, University of South Carolina College of Pharmacy, Columbia, SC, United States; ^2^Department of Population Health, Hofstra University, Hempstead, NY, United States; ^3^Department of Pharmacy Practice, Unaizah College of Pharmacy, Qassim University, Qassim, Saudi Arabia; ^4^Department of Psychiatry, Massachusetts General Hospital and Harvard Medical School, Boston, MA, United States

**Keywords:** antipsychotic medication, hospitalization, FAERS database, signal detection, atypical antipsychotics

## Abstract

**Background:**

Persons with symptoms of psychosis receiving treatment with atypical antipsychotics (AAPs) can experience serious adverse events (AEs) requiring admission to the hospital. The comparative likelihood of AE-related hospitalization following the use of all AAPs has not been fully characterized. Therefore, we evaluated the safety signals of hospitalizations associated with the use of AAPs.

**Methods:**

We conducted a cross-sectional analysis using the FDA Adverse Event Reporting System (FAERS) database (from January 1, 2004, to December 31, 2021) to examine disproportionality in reporting hospitalizations suspected to be associated with 12 AAPs (aripiprazole, asenapine, brexpiprazole, clozapine, iloperidone, lurasidone, olanzapine, paliperidone, and pimavanserin, quetiapine, risperidone, and ziprasidone). Hospitalization in the FAERs database is an outcome that is recorded as a result of an AE occurring at any drug dose. We estimated reporting odds ratios (RORs) by comparing the odds of hospitalization occurring with a particular AAP to the odds of its occurrence with other drugs. In addition, we considered the presence of a significant safety signal when the lower limit of the 95% confidence interval (CI) of the ROR is >1.

**Results:**

A total of 204,287 cases of hospitalizations were reported to the FDA for individuals treated with AAPs. There were significant safety signals of hospitalization associated with using clozapine (ROR, 2.88; 95% CI, 2.84–2.92), olanzapine (ROR, 2.61; 95% CI, 2.57–2.64), quetiapine (ROR, 1.87; 95% CI, 1.85–1.89), risperidone (ROR, 1.41; 95% CI, 1.39–1.43), aripiprazole (ROR, 1.34; 95% CI, 1.32–1.35), and ziprasidone (ROR, 1.14; 95% CI, 1.10–1.18). However, no hospitalization-related safety signals were observed with the use of paliperidone, pimavanserin, iloperidone, asenapine, lurasidone, and brexpiprazole. The ROR estimates were numerically higher among older adults than younger adults.

**Conclusions:**

This cross-sectional assessment of data from FAERs (2004–2021) suggested that users of clozapine, olanzapine, quetiapine, risperidone, aripiprazole, and ziprasidone were more likely to report being hospitalized than users of other AAPs. Given that the FAERs database only contains spontaneous reports of AEs experienced by persons exposed to a drug but without information on exposed persons who did not have an event, a cohort study comparing hospitalizations among new users of individual AAPs against each other is needed to delineate these safety signals further.

## Introduction

In patients with psychotic disorders, treatment with antipsychotic (AP) medications entails a trade-off between improving psychotic symptoms and the potential risk of adverse health outcomes requiring hospitalization (1–5). Conditions such as schizophrenia, bipolar disorder, depression, dementia, and Parkinson's disease (PD) may present with psychotic symptoms that require treatment with APs (6, 7). In these patients, hospitalization can occur due to serious adverse events (AEs) associated with AP use (4, 8–10). For example, persons treated with clozapine can experience seizures, myocarditis, pneumonia, and lifethreatening agranulocytosis and may need to be hospitalized for treatment (1, 3, 4, 11, 12). Also, persons with a clinical indication for long-term AP use who do not adhere to their medications can experience acute episodes that may necessitate admission to the hospital (13–16). Specifically, due to their relatively more favorable side effect profiles, atypical APs (AAPs) are generally preferred than typical (first-generation) APs, and they are increasingly used for a broad range of clinical indications in various psychotic disorders (17).

To help improve health outcomes and downstream expenses associated with admission to the hospital, it is crucial to compare individual AAPs and identify which among them are more likely to result in hospital admissions than others. Evidence suggests that first-generation APs were associated with a greater risk for hospitalization than AAPs (18). While there are some differences in efficacy between AAPs, their adverse effects are more different (19). Between 1989 and 2003, clozapine, risperidone, olanzapine, quetiapine, ziprasidone, and aripiprazole were the only AAPs approved by the US Food and Drug Administration (FDA). Since 2004, the FDA has approved more than 5 AAPs for diverse indications. Although some AAPs are used off-label to treat psychotic symptoms, in situations where there is no strong scientific evidence, such use can lead to AEs, and, ultimately, hospitalization (20, 21). To our knowledge, no study examined the safety signal of all AAPs related to hospitalization. Therefore, this study evaluated hospitalizations reported to the FDA associated with AAP use.

## Methods

### Data Source

We performed a cross-sectional analysis of hospitalization reports following treatment with AAPs using the publicly available data from the FDA Adverse Event Reporting System (FAERS) database. The FAERS database is a spontaneous reporting system for AEs and one of the primary tools for pharmacovigilance (22). Although the FAERs database also contains spontaneous reports data from outside the US, it is the largest and best-known national database for the surveillance of AE reports worldwide and reflects clinical practice realities. The study was exempt from ethical review because all analyzed datasets are de-identified and publicly available. In addition, we followed the Strengthening the Reporting of Observational Studies in Epidemiology (STROBE) guidelines for reporting cross-sectional studies (23).

### Primary and Subgroup Analyses

We retrieved spontaneous reports (from January 1, 2004, to December 31, 2021) of hospitalizations following the use of 12 different AAPs (aripiprazole, asenapine, brexpiprazole, clozapine, iloperidone, lurasidone, olanzapine, paliperidone, and pimavanserin, quetiapine, risperidone, and ziprasidone) from the FEARs database. Within the FAERs database, hospitalization is an outcome that is recorded as a result of an AE occurring at any drug dose. To evaluate safety signals, we examined the disproportionality in reporting hospitalizations suspected to be associated with AAP use. We estimated reporting odds ratios (RORs) by comparing the odds of hospitalization occurring with a particular AAP to the odds of its occurrence with other drugs, representing standard practice for the safety signal quantitative analyses of data in spontaneous AE reporting in similar databases (24). RORs were estimated because Rothman et al. established that estimating ROR in databases such as the FAERs is advantageous over the proportional reporting ratio (PRR), given that it estimates a relative risk (24). We considered the presence of a significant safety signal when the lower limit of the 95% confidence interval (CI) of the ROR is >1 (25). The 95% CI indicates the precision of the ROR estimate. Furthermore, to examine the influence of patient demographic variables available in the FAERs database that may likely explain a potential relationship between hospitalization and AAP use, we conducted subgroup analyses by age [older adults (65 years or older) vs. younger adults (<65 years)] and sex (male vs. female). We used SAS, version 9.4, to perform all analyses.

## Results

### Primary Findings

A total of 204,287 hospitalization cases were reported to the FDA for patients treated with AAPs (Table 1). There were significant safety signals associated with the use of clozapine (ROR, 2.88; 95% CI, 2.84–2.92), olanzapine (ROR, 2.61; 95% CI, 2.57–2.64), quetiapine (ROR, 1.87; 95% CI, 1.85–1.89), risperidone (ROR, 1.41; 95% CI, 1.39–1.43), aripiprazole (ROR, 1.34; 95% CI, 1.32–1.35), and ziprasidone (ROR, 1.14; 95% CI, 1.10–1.18). There were no significant safety signals associated with using paliperidone (ROR, 0.84; 95% CI, 0.82–0.86), pimavanserin (ROR, 0.73; 95% CI, 0.70–0.75), iloperidone (ROR, 0.71; 95% CI, 0.61–0.82), asenapine (ROR, 0.70; 95% CI, 0.66–0.74), lurasidone (ROR, 0.59; 95% CI, 0.56–0.61), and brexpiprazole (ROR, 0.34; 95% CI, 0.31–0.36).

**Table 1:** Safety signals of hospitalization associated with atypical antipsychotics use.

**AAP**	**AE reports with AAP (*n* = 642,578)**	**No. of hospitalizations (*n* = 204,287)**	**ROR**	**LL 95% CI**	**UL 95% CI**
Aripiprazole	98,129	27,119	1.34	1.32	1.35
Asenapine	8,367	1,392	0.70	0.66	0.74
Brexpiprazole	9,804	861	0.34	0.31	0.36
Clozapine	84,242	37,892	2.88	2.84	2.92
Iloperidone	1,246	210	0.71	0.61	0.82
Lurasidone	17,424	2,511	0.59	0.56	0.61
Olanzapine	80,520	34,286	2.61	2.57	2.64
Paliperidone	41,765	8,081	0.84	0.82	0.86
Pimavanserin	23,438	4,034	0.73	0.70	0.75
Quetiapine	148,278	51,525	1.87	1.85	1.89
Risperidone	110,206	31,659	1.41	1.39	1.43
Ziprasidone	19,159	4,717	1.14	1.10	1.18

*AE, adverse event; AAP, atypical antipsychotic; CI, confidence interval; LL, lower limit; UL, upper limit; ROR, reporting odds ratio*.

*A safety signal is present when the 95% confidence interval of the ROR is >1*.

### Subgroup Findings

**Figure 1:**
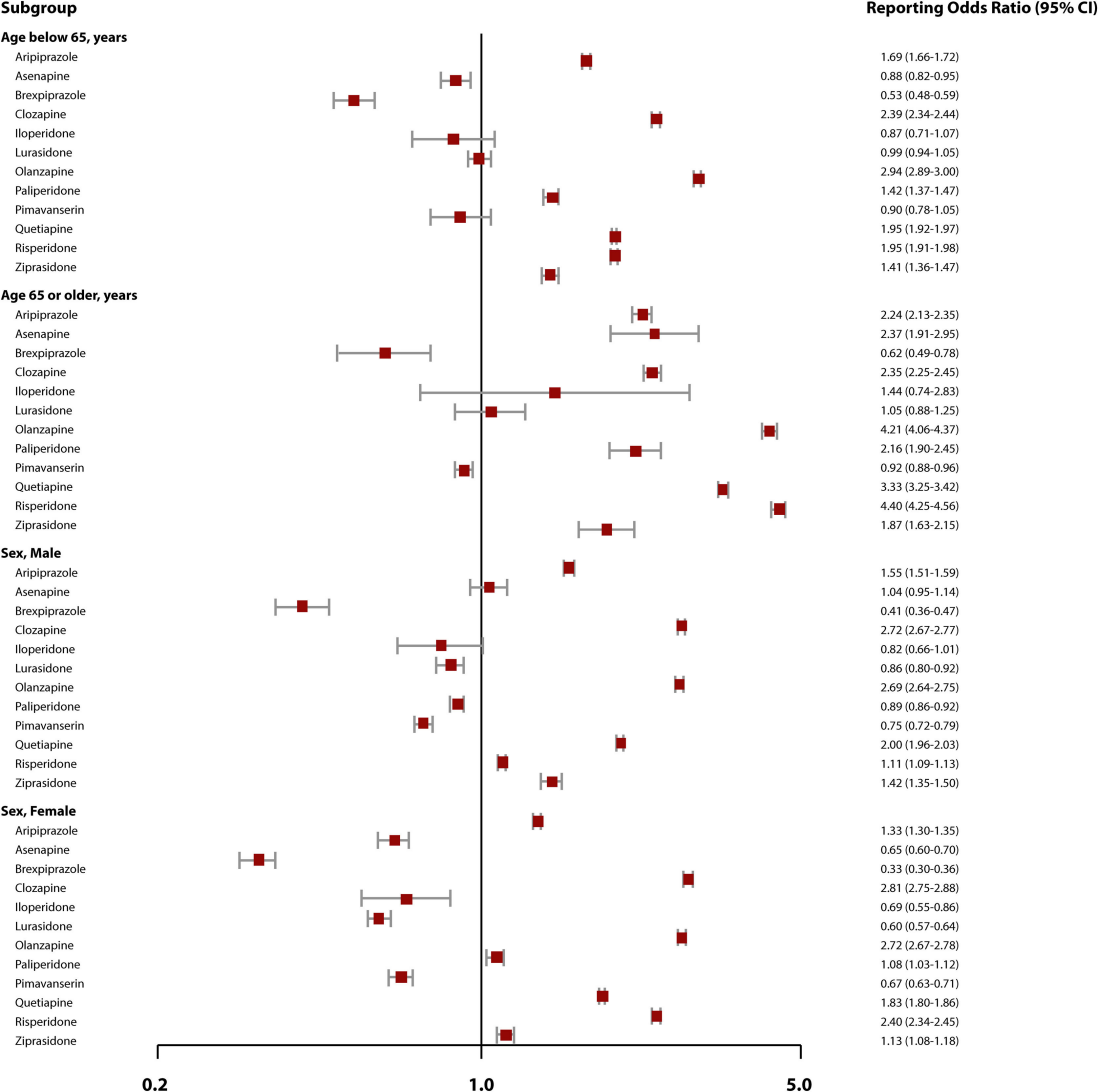
Safety signals of hospitalization associated with atypical antipsychotics use by age and sex. CI, confidence interval. A safety signal is present when the 95% confidence interval of the reporting odds ratios is >1. The maroon-colored square boxes denotes the reporting odds ratio estimates; horizontal lines represents the 95% CI.

Clozapine, olanzapine, quetiapine, risperidone, aripiprazole, and ziprasidone showed a consistent association with hospitalization across all subgroups (Figure 1). Among a subgroup of persons 65 years or older, we observed a significant safety signal for asenapine (ROR, 2.37; 95% CI, 1.91–2.95) and paliperidone (ROR, 2.16; 95% CI, 1.90–2.45). Furthermore, the estimates of hospitalization-related RORs were generally numerically higher for all AAPs among older adults than younger adults. The study also found that the reporting odds of hospitalization were significantly greater among female users of paliperidone (ROR, 1.08; 95% CI, 1.03–1.12) vs. other drugs but not among males.

## Discussion

In this cross-sectional evaluation of data from FAERs (2004–2021), we found significant safety signals related to hospitalization reports following the use of clozapine, olanzapine, quetiapine, risperidone, aripiprazole, and ziprasidone but not with paliperidone, pimavanserin, iloperidone, asenapine, lurasidone, and brexpiprazole. We also observed that asenapine and paliperidone were significantly associated with hospitalization among older adults. In addition, older adults generally had numerically higher ROR estimates for all AAPs than younger adults. Finally, the study found a hospitalization-related safety signal among female users of paliperidone but not in males.

While this study was not designed to assess the specific causes of increased hospitalization, prior research found that hospitalization following the use of AAPs is driven by several known risk factors such as age, sex, drug formulation [e.g., oral vs. long-acting injectable (LAI)], non-adherence, and living in a supervised setting (15). The current study found that older adults generally had higher RORs of hospitalization than younger adults partly because AAPs are associated with potentially serious AEs in vulnerable older adults due to age-related reduction in the ability to metabolize and excrete drugs (26). The ROR estimate for olanzapine in persons 65 years or older was almost twice that of individuals younger than 65. This remarkable difference is likely because, as a previous study demonstrated, the systemic exposure to olanzapine increases by age (27). When older adults are exposed to higher plasma concentrations of olanzapine, they can experience olanzapine-related AEs, which may necessitate being hospitalized for treatment. In addition, the study observed a significant safety signal among persons aged 65 years or above and among female users of paliperidone. These observations might be because female patients aged 65 years and above are more likely to be exposed to a higher plasma concentration of paliperidone, resulting in more AE-related hospitalizations than males (28, 29). Thus, cautious dosing when prescribing paliperidone to older females is needed (28).

The numerically higher RORs among users of olanzapine, quetiapine, risperidone, aripiprazole, and ziprasidone than lurasidone users found in this study are consistent with the results of previous cohort studies (30–33). However, while these studies were conducted in patients with schizophrenia and bipolar disorder, the FAERs data used in this study cannot accurately distinguish between the indications of various AAPs; hence we could not conduct analysis by underlying diagnoses. In addition, a retrospective cohort study of older adults with PD by Hwang et al. reported that pimavanserin was associated with an increased risk of 30-day hospitalization compared to non-use (34). However, the study did not compare hospitalizations related to other AAPs. Therefore, the implication of its findings was thought to be limited by confounding with indication due to the lack of an active comparator (34–38). Given our study's findings of hospitalization safety signals related to commonly used off-label AAPs such as quetiapine, olanzapine, risperidone, and aripiprazole (39), a cohort study comparing individual AAPs may be needed to characterize the risk of hospitalization related to different AAP use compared to each other. Such analysis should consider the clinical indication of an AAP in its design (9, 37, 38).

The fact that we did not observe safety signals among all users of AAPs approved after 2004 (paliperidone, pimavanserin, iloperidone, asenapine, lurasidone, and brexpiprazole) suggests the absence of the “*Weber effect*” (40). In this phenomenon, AE reporting peaks at the end of the second year after a regulatory authority approves a drug (41). The absence of significant safety signals might not be unrelated to the fact that, over the past decade, improvements in drug developments have contributed to having newer alternatives with limited AE profiles that result in hospitalizations (42). For example, pimavanserin has little or no D2-receptor affinity but, with a predominant 5-HT2A receptor affinity, may be helpful in PD-associated psychotic symptoms, for whom most other APs tend to worsen motor function (1). For aripiprazole, clozapine, olanzapine, quetiapine, risperidone, and ziprasidone, the hospitalization-related safety signal estimates were precise, as evident by the relatively narrow 95% CI of the RORs. This observation might be related to the fact that they are more frequently used AAPs across wide-ranging clinical indications throughout the study period (2004–2021). Thus, they received more reports of AE-related hospitalizations. While clinicians are encouraged to avoid using new drugs when older, similarly efficacious alternatives are available (43), individualized consideration of the likelihood that using a drug results in AE-related hospitalization will help improve the health outcomes of patients with a clinical indication for AAPs.

### Study Limitations

This study's findings should be interpreted while considering the following limitations. First, signal detection methods employed in the current study can only identify potential drug safety risks but not rule them out. This is because spontaneous reports to FAERs only contained information on persons exposed to a drug and had an AE but with no information about persons who took the drug and did not experience an event. Second, due to its passive surveillance nature and reporting fatigue among clinicians and patients, the FAERS database is subject to underreporting (44). Third, the study could not compare AAPs by formulations and indications. With the established evidence that the use of LAI AAPs was associated with a lower risk of hospitalizations than orals (45), the study would have estimated this possible difference in a subgroup analysis if we had this information at our disposal. Fourth, the study could not estimate the time from exposure to a drug and incidence of hospitalization because such information cannot be estimated reliably using the FAERs data. Fifth, since hospitalization is recorded as a result of an AE occurring at any drug dose in the FAERs database, we were unable to provide information regarding different doses of AAPs. Finally, the study wasn't able to provide information on the specific type of AEs that resulted in hospitalization. Despite these limitations, spontaneous reports represent a valuable tool to monitor potential new safety signals concerning AAPs.

## Conclusions

In this cross-sectional assessment of data from the FAERs, users of clozapine, olanzapine, quetiapine, risperidone, aripiprazole, and ziprasidone were significantly more likely to report being hospitalized than users of other AAPs. However, given that the FAERs database is limited by having information on spontaneous reports of persons exposed to a drug and experienced an AE, but without information on those who did not have the event, a cohort study comparing the risk of hospitalization among users of individual AAPs against each other is needed to further delineate these safety signals.

## Data Availability Statement

Publicly available datasets were analyzed in this study. This data can be found here: https://www.fda.gov/drugs/questions-and-answers-fdas-adverse-event-reporting-system-faers/fda-adverse-event-reporting-system-faers-public-dashboard.

## Ethics Statement

The study was exempt from ethical review by the University of South Carolina Institutional Review Board because all analyzed datasets are de-identified and publicly available.

## Author Contributions

IY and CT: drafting and revision of the manuscript for content, including medical writing for content, major role in the acquisition of data, study concept or design, and analysis or interpretation of data. IK, EC, SA, and NM: drafting and revision of the manuscript for content, including medical writing for content, and study concept or design. All authors contributed to the article and approved the submitted version.

## Conflict of Interest

The authors declare that the research was conducted in the absence of any commercial or financial relationships that could be construed as a potential conflict of interest.

## Publisher's Note

All claims expressed in this article are solely those of the authors and do not necessarily represent those of their affiliated organizations, or those of the publisher, the editors and the reviewers. Any product that may be evaluated in this article, or claim that may be made by its manufacturer, is not guaranteed or endorsed by the publisher.
